# Bare necessities? The utility of full skin examination in the COVID‐19 era

**DOI:** 10.1111/ced.14620

**Published:** 2021-03-18

**Authors:** C. O’Connor, C. Gallagher, M. O’Connell, J. Bourke, M. Murphy, M. Bennett

**Affiliations:** ^1^ Department of Dermatology South Infirmary Victoria University Hospital Cork Ireland; ^2^ Department of Medicine University College Cork Cork Ireland

## Abstract

Full skin examination (FSE) may improve the detection of malignant melanoma (MM). The objective of this study was to assess the safety of targeted lesion examination (TLE) compared with FSE in our Pigmented Lesion Clinic (PLC). Patients attending the PLC were randomized in a 2 : 1 ratio to FSE (intervention) or TLE (standard care). Demographic details and risk factors were documented, and the time taken to perform FSE and TLE was noted. Of 763 participants, 520 were assigned to FSE and 243 were assigned to TLE. On average, FSE took 4.02 min and TLE took 30 s to perform. Of the 520 participants assigned to FSE, 37 (7.1%) had incidental findings, of whom 12 patients (2.3%) had additional lesions biopsied. No additional melanomas were detected that would have been missed by use of the standard protocol. This study suggests that in low‐risk patients referred to a PLC with a lesion of concern, the possibility of missing incidental cutaneous malignancies using lesion‐directed examination is low.

Full skin examination (FSE) may help in the early detection of malignant melanoma (MM).^1^ However, the US Preventative Services Task Force does not recommend routine FSE, citing a lack of evidence for its efficacy in reducing mortality.^2^ Hartman *et al*. recently highlighted the economic and time limitations in routinely performing FSE.^3^ The monumental impact of the COVID‐19 pandemic has increased pressure on dermatology resources, and patient exposure to unnecessary healthcare interventions should be minimized.^4^

The Pigmented Lesion Clinic (PLC) at our centre accepts referrals from general practitioners (GPs) for patients with a suspicious pigmented lesion(s). The lesion(s) in question is examined by a consultant dermatologist. Any other lesion of concern to the patient is also examined. FSE is performed only in patients with a prior personal or family history of MM and in male patients aged > 50 years, as these patients are considered to be at higher risk of MM. Patients who have a suspicious lesion biopsied also undergo FSE.

The aim of this study was to assess the safety of targeted lesion examination (TLE) compared with FSE, and to determine the types of lesions that were likely to be missed if only TLE, and not FSE, was performed.

## Report

Ethics approval was provided by the Clinical Research and Ethics Committee of the Cork Teaching Hospitals. Patients attending 34 PLCs over a 20‐month period were invited to participate in the study, and written informed consent was obtained.

Patients were randomized in a 2 : 1 ratio to either FSE (intervention group) or TLE (standard of care). Male patients aged > 50 years and patients with a personal or family history of MM were assigned to FSE as per departmental protocol. Those selected to undergo FSE had the additional examination performed by a senior dermatology specialist registrar, following their standard assessment (TLE) by a consultant dermatologist. Those randomly selected to undergo TLE had the examination performed by a consultant dermatologist as part of the standard assessment.

Demographic details recorded included age, sex, personal or family history of MM, prior sunbed use, average number of sun holidays per year, prolonged periods spent abroad and occupation (indoor, outdoor or mixed). The length of the examination was timed for both FSE and TLE, and details of additional identified lesions were recorded. A biopsy was taken from any patients in whom additional suspicious lesions were identified.

In total, 766 patients consented to participate; 523 of these patients were assigned to FSE and 243 were assigned to TLE. Three patients declined FSE, citing reasons such as time pressure, embarrassment, and menstruation, resulting in a final group size of 520 for FSE. No patient declined TLE. Most participants were white Irish, female, middle‐aged and worked indoors; 1.3% had a previous history of MM, 7.9% had a family history of MM and 25.6% had previously used sunbeds (Table 1).

**Table 1:** Patient demographics with details on melanoma history and sun exposure.

Full skin examination (*n* = 520)	Result
Demographics	
Female sex, *n* (%)	372 (71.5)
Average age, years	44.9
White Irish, *n* (%)	479 (92.1)
MM history, *n* (%)	
Personal history of MM	7 (1.3)
First‐degree relative with MM	24 (4.6)
Second‐degree relative with MM	17 (3.3)
Two or more relatives with MM	4 (0.8)
Sun exposure history, *n* (%)	
Indoor occupation	405 (77.9)
Outdoor or mixed occupation	115 (22.1)
Previous sunbed use	133 (25.6)
Previous phototherapy	1 (0.2)
Migration > 12 months outside Ireland	85 (16.3)

MM, malignant melanoma.

On average, FSE took 4.02 min to perform, from asking the patient to undress to the patient being fully dressed following the examination. By contrast, the average TLE took 30 s, therefore FSE took eight times longer than TLE. FSE took slightly longer (5.6 min) in male patients aged > 60 years, whereas the duration of TLE was not dependent on age or sex.

Of the 520 patients assigned to FSE, 37 (7.1%) had incidental findings on FSE, including inflammatory dermatoses such as psoriasis and eczema, and previously undiagnosed porphyria cutanea tarda (Table 2). Twelve patients (2.3%) had additional lesions biopsied, including a superficial basal cell carcinoma. No additional MMs were detected by FSE that would otherwise have been missed by use of the standard protocol (TLE).

**Table 2:** Additional clinical or histological diagnoses detected on full skin examination that would not have been detected with targeted lesional examination, excluding seborrhoeic keratosis, actinic keratosis, lentigines, viral warts, angiomata, acne, folliculitis and keratosis pilaris.

Diagnoses	*n* (%)
Clinical diagnosis	
Dermatofibroma	10 (1.9)
Psoriasis	6 (1.2)
Dermatitis	4 (0.8)
Naevus spilus	4 (0.8)
Linear epidermal naevus	2 (0.4)
Porphyria cutanea tarda	2^a^ (0.4)
Pigmented purpuric dermatosis	2 (0.4)
Halo naevus	1 (0.2)
Becker naevus	1 (0.2)
Lichen simplex chronicus	1 (0.2)
Hidradenitis suppurativa	1 (0.2)
Port wine stain	1 (0.2)
Erythema ab igne	1 (0.2)
Varicose ulcer	1 (0.2)
Histological diagnosis	
Dysplastic naevus with mild atypia	10 (1.9)
Blue naevus	1 (0.2)
Basal cell carcinoma, superficial	1 (0.2)

^a^ Only one attended for confirmatory biochemical diagnosis.

This study suggests that in low‐risk patients referred to a PLC with a lesion of concern, the possibility of missing incidental cutaneous malignancies using lesion‐directed examination is low, and that TLE is a safe and efficient practice in this setting. A previous study has shown that most MMs are picked up in patients referred with a lesion rather than during routine mole checks.^5^ Patients attending our PLC have already been reviewed by their GP and therefore any lesions of concern may have already been identified by FSE in primary care. Dermatologists have also reported patient embarrassment and time constraints as significant barriers to skin cancer screening using FSE.^6^, ^7^, ^8^ In this study, at the time of randomization, three patients declined participation in FSE, but none declined participation in TLE, suggesting that patient satisfaction with TLE is high.

There may be limitations to extrapolating these data to other regions with higher prevalences of MM, such as Australia and New Zealand. One Australian study showed a high rate of MM detected incidentally by FSE in private practice, mostly in men, with a mean age of 61.9 years.^7^ Dermatology training may also be affected if TLE is adopted over FSE, as trainees may have less experience in assessing benign or low‐risk lesions such as seborrhoeic keratoses, angiomas or benign melanocytic naevi.

Access to specialist dermatologist services is limited, particularly in the COVID‐19 era. This study shows that FSE takes eight times longer than TLE. Although FSE is superior to TLE if resources are unlimited, FSE has minimal benefit in detecting additional skin cancers and TLE permits more patients to be seen in our restricted face‐to‐face (F2F) clinic appointments. Therefore, we suggest that TLE should be adopted as the standard of care for low‐risk patients in PLC in order to optimize efficiency in the COVID‐19 era.

**Learning points**

- FSE may help in the early detection of MM; however, FSE may not reduce mortality, and has economic and time limitations.
- The COVID‐19 pandemic has increased pressure on dermatology resources, and patient exposure to unnecessary healthcare interventions should be minimized.
- This study suggests that in low‐risk patients attending a PLC referred with a lesion of concern, the possibility of missing incidental cutaneous malignancies using TLE is low.
- Although FSE is superior to TLE if resources are unlimited, FSE has minimal benefit in detecting additional skin cancers, and TLE permits more patients to be seen in our restricted F2F clinic appointments.
- TLE should be adopted as the standard of care in PLC to optimize efficiency in the COVID‐19 era.
